# A Rapid Subtractive Immunization Method to Prepare Discriminatory Monoclonal Antibodies for Food *E. coli* O157:H7 Contamination

**DOI:** 10.1371/journal.pone.0031352

**Published:** 2012-02-07

**Authors:** Min Jin, Jing Lang, Zhi-Qiang Shen, Zhao-Li Chen, Zhi-Gang Qiu, Xin-Wei Wang, Jun-Wen Li

**Affiliations:** Department of Environment and Health, Institute of Health and Environmental Medicine, Key Laboratory of Risk Assessment and Control for Environment and Food Safety, Tianjin, China; National Cancer Institute, United States of America

## Abstract

To detect food *E. coli* O157:H7 contamination rapidly and accurately, it is essential to prepare high specific monoclonal antibodies (mAbs) against the pathogen. Cyclophosphamide (Cy)-mediated subtractive immunization strategy was performed in mice to generate mAbs that react with *E. coli* O157:H7, but not with other affiliated bacteria. Specificity of 19 mAbs was evaluated by ELISA and/or dot-immunogold filtration assay (DIGFA). Immunogloubin typing, affinity and binding antigens of 5 selected mAbs were also analysed. MAbs 1D8, 4A7, 5A2 were found to have high reactivity with *E. coli* O157:H7 and no cross-reactivity with 80 other strains of bacteria including *Salmonella sp.*, *Shigella sp*., *Proteus sp.*, *Yersinia enterocolitica*, *Staphylococcus aureus*, *Klebsiella pneumoniae*, *Citrobacter freundii* and other non-*E. coli* O157:H7 enteric bacteria. Their ascetic titers reached 1∶10^6^ with *E. coli* O157:H7 and affinity constants ranged from 1.57×10^10^ to 2.79×10^10^ L/mol. The antigens recognized by them were different localized proteins. Furthermore, immune-colloidal gold probe coated with mAb 5A2 could specifically distinguish minced beef contaminated by *E. coli* O157:H7 from 84 other bacterial contaminations. The Cy-mediated subtractive immunization procedure coupled with hybridoma technology is a rapid and efficient approach to prepare discriminatory mAbs for detection of *E. coli* O157:H7 contamination in food.

## Introduction

*E. coli* O157:H7, a species of enterohemorrhagic *E. coli*, is the bacterium that causes diarrhea. *E. coli* O157:H7 outbreaks have become a severe threat to human health. In 1993, more than 700 people in the USA were infected with *E. coli* O157:H7 contaminated Jack in the Box hamburgers. In August 1997, Hudson Foods, a major hamburger supplier for Burger King, recalled 35 million pounds of ground beef (the largest food recall in the nation's history), as a result of a major *E. coli* O157:H7 outbreak (http://www.downtoearth.org/health/general-health/rise-food-poisoning-america). In 1996, the most serious *E. coli* O157:H7 infection in the world occurred in Japan and resulted in 10 deaths and more than 9,000 sick people [1]. Up to now, it has become one of the most important pathogens that caused food borne diseases. Therefore, it is necessary to develop rapid, sensitive and specific methods to detect *E. coli* O157:H7 in clinical or food samples without further tedious and time consuming cultivation of the bacterium. Immunological diagnostic methods, which utilize specific antibody, were under consideration due to their simple and rapid protocols. However, their efficacies mainly depend on the quality of the specific monoclonal antibodies (mAbs). Ideally, mAbs selected for use in *E. coli* O157:H7 detection should have no cross-reactivity with other enterobacteria. However, *E. coli* O157:H7 share some common structural epitopes in its lipopolysaccharides with *Salmonella* group N, *Yersinia enterocolitica* and other enterohemorrhagic *E. coli*, which may result to cross-reactions for *E. coli* O157:H7 mAbs [2], [3]. Sowers *et al.* investigated the specificity of antisera for O157 and H7 referred by the Centers for Disease Control and Prevention (CDC) and demonstrated its reaction with a strain of *Citrobacter freundii* and all four strains of *Salmonella* O group N (O30) [4]. Therefore, it is difficult to obtain a high specific antibody against *E. coli* O157:H7.

Subtractive immunization (S.I.) is a proven technique to prepare mAbs specific for antigens that are present in low abundance in a protein mixture, poorly immunogenic and/or in similar structure or sequence with other proteins [5]. It is generally performed through a distinct immune tolerization approach that uses the immunosuppressive agent Cyclophosphamide (Cy) coupled with immunizations with two phenotypically distinct cell or protein variants as sequential immunogens. This procedure not only eliminates the production of undesired mAbs, but also achieves a highly specific antibody and increases the likelihood of generating mAbs against rare or weakly immunogenic epitopes in complex biological mixtures such as intact cells or tissues. There are several unique reactive antibodies targeting special protein or cells prepared successfully by this technique [6], [7], [8], [9]. However, to the best of our knowledge, there is so far no report on the preparation of discriminatory mAbs for bacteria by this procedure.

In the present article, we have generated a pool of mAbs by the S.I.-hybridomas procedure. The mice were first immunized with *E. coli* O157:H19, and subsequently with *E. coli* O157:H7. The first and second immunizations were intervened by treatment with the immunosuppressant drug, Cy. With this technique, we prepared 3 specific mAbs exhibiting no cross-reactivity with other non-*E. coli* O157:H7 targets. Compared with traditional hybridoma technology, it is a rapid and efficient approach to prepare discriminatory mAbs for the detection of *E. coli* O157:H7 in food.

## Materials and Methods

### Ethics Statement

All animal procedures involving the care and use of animals were in accordance with the regulations concerning the ethics of science research in the Institute of Health and Environmental Medicine and approved by the Ethics Review Board of Institute of Health and Environmental Medicine (protocols #JKYSS-2007-002 and #JKYSS-2007-003).

### Bacterial Strains

The bacteria (listed in Table 1) grew on Trypticase soy agar (TSA; BD Co.) or nutrition agar (NA; BD Co.) plates at 37↓°C overnight. All the standard strains were purchased from China Medical Culture Collection (CMCC) or American Type Culture Collection (ATCC). Some other E. coli strains which included serotypes O26:H11 (IHEM 1.3035), O50:H7 (IHEM 1.3036), O111:H8 (IHEM 1.3037), and O145:NM (IHEM 1.3038) were isolated from patients with hemolytic uremic syndrome or hemorrhagic colitis and deposited in the Microbiological Culture Collection Center of the Institute of Health and Environmental medicine (IHME, Tianjin, China). Whole-cell antigens were prepared using a previously described method [10].

**Table 1:** Bacterial strains used in the study.

Bacterial strain	Medium and growth temp	No. of isolates tested	Collection or isolation source^a^ (serotype)
*Escherichia coli*	TSA, 37°C	26	
Non O157		21	CMCC 44102, 44109, 44110, 44113, 44127(O14), 44149 (O127a:K63), 44155(O111:K58), 44156(O111:K58:H2), 44186(O26:K60), 44216(O14), 44505(O19b:H7), 44561(O128:K67:H2), 44813(O78:H11), 44824(O15:H21), 44825(O143), 44336(O55:K59:H6), 44338(O86a:K61);IHEM 1.3035(O26:H11),1.3036(O50:H7),1.3037(O111:H8),1.3038(O145:NM).
O157:H19		1	CMCC 44752
O157:H7		4	ATCC 43895; IHEM 1.3001,1.3002,1.3003.
*Salmonella sp.*	TSA, 37°C	26	CMCC 50001, 50041, 50042, 50058, 50061, 50062, 50065, 50067, 50115, 50136, 50303, 50306, 50309, 50312, 50313, 0315, 50322, 50325, 50327, 50770, 50774, 50781, 50798, 50825, 50885, 50866
*Shigella*	TSA, 37°C	18	CMCC 51510, 51512, 51513, 51514, 51515, 51516, 51517, 51518, 51519, 51521, 51522, 51524, 51361, 51499, 51135, 51066, 51067, 51081
*Staphylococcus aureus*	NA, 37°C	4	CMCC 26003, 26071, 26113, 29213
*Yersinia enterocolitica*	TSA, 37°C	4	CMCC 52215, 52216, 52219, 52243
*Citrobacter freundii*	NA, 37°C	1	IHEM 1.5001
*Klebsiella pneumoniae*	NA, 37°C	2	CMCC 46012; IHEM 1.6001
*Proteus vulgaris*	NA, 37°C	1	CMCC 49001
*Proteus morganii*	NA, 37°C	1	CMCC 49087
*Proteus mirabilis*	NA, 37°C	1	CMCC 49106

a : CMCC, China Medical Culture Collection; ATCC, American Type Culture Collection; IHEM, Microbiological Culture Collection Center which belonged to the Institute of Health and Environmental Medicine (Tianjin, China); TSA, Trypticase soy agar; NA, nutrition agar.

### Subtractive Immunization Procedure

Six 5-week-old BALB/c mice were obtained from the Experimental Animal Center in the Academy of Military Medical Sciences (Beijing, China). The S.I. procedure was performed as follows according to Sleister and Rao's protocol [5]. Briefly, on day 1, 10^8^ CFU *E. coli* O157:H19 (CMCC 44752) as tolerogen were injected (intraperitoneal, i.p.). Cy (Sigma, USA) was injected (100 mg per kg, i.p.) at 10 min, 24 hr and 48 hr later. The *E. coli* O157:H19 and Cy injections cycle was repeated three other times throughout a 14-day interval. On day 55, a tail bleed were collected to detect Ab reactivity to *E. coli* O157:H19 in whole cell ELISA. Once the mice had obtained the suppression capacity to the antigenic determinants of the *E. coli* O157:H19, the mice were further immunized as follows: 10^8^ CFU *E. coli* O157:H7 (ATCC 43895) was injected (i.p.) intraperitoneally on day 55, 65, 72 and 86. On day 89, mice were sacrificed by cervical dislocation and blood was collected through the orbital sinus after eyeball extirpation. Spleen cells were harvested for the preparation of hybridomas. In addition, five litter mate control mouse received the same series of injections, except that the Cy-mediated tolerance procedure was omitted.

### Construction of Hybridoma Cells

Fusions of Sp2/0 myeloma cells (China Center for Type Culture Collection, CCTCC) with spleen cells from above immunized BALB/c mice were performed according to Lane *et al.* [11]. Supernatants from wells with hybridoma growth were screened for the production of antibodies against *E. coli* O157:H7 and *E. coli* O157:H19 by the indirect ELISA described below. Positive hybridoma cultures were cloned by two cycles of cloning in limited dilution.

### Production of mAbs

mAbs were produced by the *in vitro* and *in vivo* propagation of hybridoma cells. *In vitro* cells were propagated in the RPMI-1640 medium (Gibco, USA) with 10% FBS. Harvested media were centrifuged at 3,000 *g* for 30 min. Then, the supernatants were collected and stored at −70°C. *In vivo* cells were propagated in 8-week-old BALB/c mice to generate ascites fluid. The operating procedure was carried out in accordance with *Guidelines for Monoclonal Antibody Production 2008* (NHMRC, http://www.ag.gov.au/cca) and the ascites fluid was stored at −70°C until purification.

### Purification of mAbs

Following sequential precipitation with caprylic acid and ammonium sulphate [12], the Protein A HP SpinTrap (GE healthcare, USA) was used to purify mAbs from ascitic fluid and cell culture supernatants according to the manufacturer's instructions.

### Indirect ELISA

Indirect ELISA was used to assess the mAbs responses to various bacteria [13]. To observe the specificity of the mAbs, 10^6^ cfu of various tested bacteria diluted in carbonated-bicarbonate buffer (50 mmol/L, pH 9.6) were added to 96-well microtiter plates (Nunc, Denmark) and allowed to coat wells overnight at 4°C. To observe the sensitivity of the mAbs, the 96-well microtiter plates were coated with 3×10^1^–3×10^7^ cfu *E. coli* O157:H7 diluted in carbonated-bicarbonate buffer (50 mmol/L, pH 9.6) and allowed to coat wells overnight at 4°C. Then, the plates were washed three times with PBS containing 0.05% Tween 20 (PBS/T) and blocked with PBS containing 1% bovine serum albumin (Sigma, USA) for 1 h at 37°C. Following washing three times, mAbs at various serial dilutions or PBS (negative control) were added to each well and incubated for 1 h at 37°C. Plates were washed with PBS/T three times and then incubated for 1 h at 37°C with horseradish peroxidase (HRP)-labeled rabbit anti-mouse IgG (1∶50000, Abcam, USA). After washing, substrate solution (3,3′,5,5′-tetramethybenzidine) was added and the plate was incubated at room temperature for 30 min. The colorimetric reaction was interrupted with 2 mol/L sulphuric acid and the optical density (OD) was recorded at 450 nm. A test sample was considered positive if the ratio (T/C) of the OD value in the test well (T) to that of the negative control well (C) was ≥2.1.

### mAbs Isotyping

mAbs isotypes were determined using the Monoclonal antibody Isotyping Kit I (Sigma, USA) by using indirect ELISA in which 10^6^ cfu *E. coli* O157:H7 was coated on the 96-well microtiter plates. The protocol was performed according to the manufacturer's instructions.

### Functional Affinity Constant of mAbs

The functional affinity constant of mAbs was determined according to Loomans *et al*. [14] and the antibody dilutions resulting in 50% of the maximum absorbance value at a particular coating concentration were selected for the affinity calculation. It was calculated according to Beatty *et al*. [15].

### Western Blot Analysis

The recognized antigens of the mAbs were analyzed by western blotting as previously described [13]. Briefly, fifty micrograms of *E. coli* O157:H7 cell lysates (in 50 mmol/L Tris–HCl, pH 7.4, 1% Triton X-100, 150 mmol/L NaCl, 1 mmol/L EDTA) were separated by 10% SDS-PAGE, then transferred onto a nitrocellulose membrane. Membranes were blocked with PBS containing 5% skimmed milk powder and 0.1% Tween 20 for 1 h at room temperature. After washing, the membranes were placed into a MiniProtean Multiscreen Apparatus (GE healthcare, USA). Supernatants of different anti- *E. coli* O157:H7 cell-specific mAbs were diluted 1∶4 in blocking buffer and added to separate channels in the apparatus, and incubated for 1 h at room temperature. After five washes with 0.1% Tween20/PBS, membranes were then incubated with HRP–conjugated goat anti-rabbit IgG (1∶3000, Abcam, USA) for 1 h at room temperature. After a further five washes with 0.1% Tween 20/PBS, bound conjugate was detected by Enhanced Chemiluminescence Reagent (GE healthcare, USA).

### Preparation of Colloidal Gold Probe

The gold-labeled anti-*E. coli* O157:H7 mAbs conjugate was prepared following the procedure described by Wang *et al.* [16]. The dispersion of colloidal gold particles was obtained by heating a solution containing 0.01% AuCl_3_·HCl·4H_2_O (Sigma, USA) and 0.1% sodium citrate (Sigma, USA). The pH of the dispersed gold solution was adjusted to 8.3 with 2% K_2_CO_3_ after the solution had cooled. 150 µL of the purified anti-*E. coli* O157:H7 mAb (0.5 g/L) were added to 10 mL colloidal gold solution. The mixture was slowly stirred for 10 min then incubated overnight at 4°C without stirring. The reaction mixture was centrifuged at 10,000×g for 60 min, 4°C and the precipitate was suspended in 1 mL 0.01 mol/L PBS/T (pH 7.4) containing 1% BSA, 0.02% NaN_3_ and 0.5% Tween-20.

### Preparation of Food Samples Contaminated by Various Pathogens

Minced beef was used as the food sample for examination of various bacteria contaminations as described by Fukushima with modifications [17]. Each 25 g minced beef and 10^9^ CFU bacterial solution listed in the Table 1 were mixed with 225 mL of 0.02% Tween 20-buffered peptone water (Tween20-BPW) and homogenized in a stomacher 400 Circulator (Seward, English) for 1 min. We added Tween 20 to BPW to emulsify fat in the samples. Approximately 220 mL of filtration of the homogenates was removed and then was centrifuged at 1,880 g for 5 min at room temperature. The supernatant was transferred into a new tube and then centrifuged at 16,000 g for 5 min at room temperature. The final pellet was suspended in 1.5 mL of 0.15 mol/L NaCl and used for sampling.

### Dot-Immunogold Filtration Assay (DIGFA)

To rapidly detect *E. coli* O157:H7 in food samples, DIGFA, a solid phase labelled immunoassay technique that uses a nitrocellulose membrane (NCM) as a support and colloidal gold as the label was performed according to Han *et al.* with modifications [18]. Firstly, the NCM (Millipore, USA) was prepared by soaking it with triple-distilled water and then air dried. The disc, 2 cm in diameter, was made from the prepared membrane with a punch and soaked in 0.05 mol/L carbonate buffer and then air-dried. Secondly, E. coli O157:H7 was detected by dripping 2 µL of the minced beef sample onto the disc. After this was air-dried, a 2 µL of blocking agent (0.01 mol/L PBS containing 1% BSA, pH 7.4) was added, followed by the addition of 5 µL of colloidal gold probe. Finally, 10 µL of lotion (0.01 mol/L PBS containing 0.05% Tween-20, pH 7.4) was added. Results were assessed by the appearance of a reddish dot in the opening indicating a positive reaction, whereas the absence of such a dot indicated a negative reaction. A total of 2 µL of goat anti-mouse IgG replacing testing samples was used as the internal control to prove efficacy of the colloidal gold probe. The appearance of a reddish dot suggested that this colloidal gold probe was indeed effective.

## Results

### Immune Suppression of *E. coli* O157:H19 by Cy Treatment

**Figure 1:**
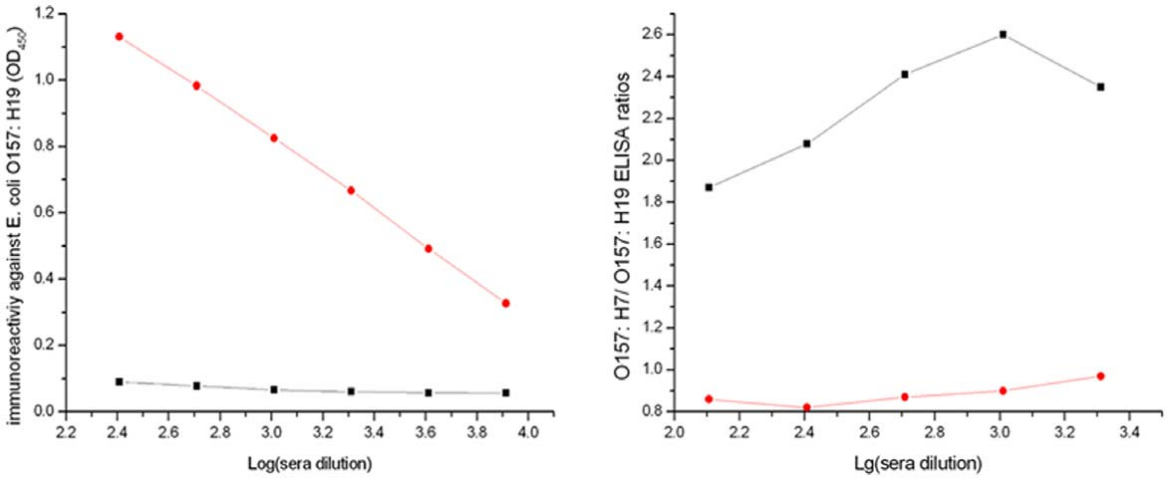
Immune suppression of *E. col*i O157:H19 on day 55 and enhancement of immune response to *E. coli* O157:H7 on day 89 by Cy-mediated subtractive immunization procedure. A, sera from Cy-treated mice (experimental group) and no Cy-treated mice (control group) were diluted and tested for antibody reactivity to *E. coli* O157:H19 in whole cell ELISA (OD 450 nm); B, sera were diluted and tested for reactivity to *E. coli* O157:H7 and *E. coli* O157:H19 (▪: experimental group; •: control group).

The Cy-mediated S.I. strategy was used to suppress the immune system of mice to the highly reactive immunodeterminants present on *E. coli* O157:H19 that was inoculated as the initial immunogen (experimental group). Sera from control mouse group (no Cy treatment) exhibited a high antibody titer (about 1∶10^4^) with the *E. coli* O157:H19, while the sera from mice treated with Cy showed negligible or no detectable reactivity on the *E. coli* O157:H19 (Fig. 1A). Therefore, this indicated the mice had obtained the suppression capacity to the antigenic determinants of the *E. coli* O157:H19.

### Enhancement of Immune Response to *E. coli* O157:H7

On day 89, sera were collected and screened for reactivity to *E. coli* O157:H7 and *E. coli* O157:H19 with indirect ELISA. The control mice demonstrated higher antibody titers to *E. coli* O157:H19 than to *E. coli* O157:H7 as indicated by low O157:H7/O157:H19 ELISA ratios (Fig. 1B). On the contrary, the sera from the mouse which had been treated with Cy after *E. coli* O157:H1_9_ inoculation, showed much lower reactivity towards *E. coli* O157:H19 than to *E. coli* O157:H7 as their O157:H7/O157:H19 ELISA ratio can reached up to 2.6. These data indicate that Cy suppressed the immune response to *E. coli* O157:H19 sharply and permitted an enhanced differential response to *E. coli* O157:H7.

### Antigen Specificity and Sensitivity of mAbs from Experimental Group

Nineteen hybridoma clones were generated from the above experimental group mice after screening by indirect ELISA and three times of subcloning, which were named as 1C6, 1D8, 1H6, 3F5, 3G12, 4A7, 4D7, 5A2, 5A5, 5A9, 5C12, 5D8, 5F10, 5F11, 5H4 , 6C11, 6D12, 7F1 and 7F8, respectively. The target antigen specificity of mAbs from above selected clones was measured by indirect ELISA with 84 strains of bacteria. mAbs from *in vivo* cells of 1C6, 1D8, 1H6, 4A7, 5D8, 5A2 clones demonstrated good quality, which had a titer of 1∶2048 to *E. coli* O157 and negligible reactivity towards other tested food-born pathogens including *Salmonella sp.*, *Shigella sp*., *Proteus sp.*, *Yersinia enterocolitica*, *Staphylococcus aureus*, *Klebsiella pneumoniae*, *Citrobacter freundii* and other non-*E. coli* O157 enteric bacteria (Table 2). Above all, mAbs 1D8, 4A7, 5A2 produced from hybridomas in mouse ascites had a high titer with *E. coli* O157:H7 (1∶10^6^), and showed no cross-reactivity with all the other tested bacteria. In additions, their sensitivity to *E. coli* O157:H7 can reach to 3×10^4^ cfu/mL. However, mAbs from other clones including 3F5, 3G12, 4D7, 5A5, 5A9, 5C12, 5F10, 5F11, 5H4, 6C11, 6D12, 7F1 and 7F8 were showed to be unspecific and react with several other non-targets (Table 2 and data not shown). We also compared the specificity of our new antibodies with a commercially available antibody against *E. coli* O157:H7, the ab75244 from the Abcam, USA. The following strains were tested including *Salmonella sp.* (CMCC 50325), *Citrobacter freundii* (IHEM 1.5001), *Staphylococcus aureus* (CMCC 29213), *Shigella* (CMCC 51081,51066), Non-O157 *E. coli* (CMCC 44216, 44505), *E. coli* O157:H19 (CMCC 44752), *E. coli* O157:H7 (ATCC 43895). The ab75244 (1 mg/mL) exhibited high antibody titers (about 1∶16384) with both the *E. coli* O157:H7 and the E. coli O157:H19. No cross-reactivity was found with other strains. Therefore, the ab75244 is not able to discriminate *E. coli* O157:H7 from *E. coli* O157:H19, whereas our new mAbs 1D8, 4A7, 5A2 prepared by subtractive immunization distinguish *E. coli* O157:H7 from *E. coli* O157:H19 effectively.

**Table 2:** Summary of the characteristic of some mAbs reacting with *Escherichia coli* O157:H7 and others.

Organisms	No of strains tested	Positive strain number to be detected by mAbs							
		1C6	1D8	4A7	5A2	5D8	1H6	3G12	4D7
*Escherichia coli*	26								
O157:H7	4	4	4	2	4	4	4	4	4
O157:H19	1	1	0	0	0	1	1	1	0
*Non*-O157	21	0	0	0	0	0	0	4	5
*Salmonella sp.*	26	0	0	0	0	0	0	2	6
*Shigella sp.*	18	0	0	0	0	0	0	0	3
*Proteus spp.*	3	0	0	0	0	0	0	0	0
*Yersinia enterocolitica*	4	0	0	0	0	0	0	0	0
*Staphylococcus aureus*	4	0	0	0	0	0	0	2	1
*Klebsiella pneumoniae*	2	0	0	0	0	0	0	0	0
*Citrobacter freundii*	1	0	0	0	0	0	0	0	0

### Determination of Immunoglobulin Types and Functional Affinity Constant of mAbs

The immunoglobulin typing of mAbs 1C6, 1D8, 4A7, 5A2 and 5D8 showed that the immunoglobulin subclass was IgM-type for mAbs 1C6, 5A2, 5D8 clones and IgG type for 4A7 and 1D8 (Table 3). Moreover, based upon the analytical curve of affinity for various mAbs, the mean Kaff for 5 mAbs ranged from 1.57×10^10^ to 1.47×10^11^ L/mol as listed in the Table 3.

**Table 3:** The characteristics of mAbs against *E. coli* O157:H7.

mAbs	Sub-class type	Affinity constant (L/mol)
1C6	IgM	1.90×10^10^
1D8	IgG1	1.57×10^10^
4A7	IgG2b	2.79×10^10^
5A2	IgM	1.47×10^11^
5D8	IgM	1.83×10^10^

### Properties of the Antigens Recognized by the mAbs

**Figure 2:**
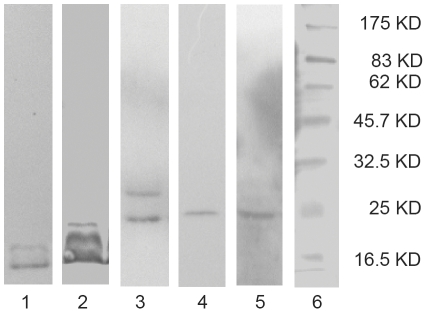
Protein antigens are detected by Western blot analysis. *E. coli* O157:H7 cell lysates were subjected to SDS–PAGE, transferred to nitrocellulose filter, and probed with 1C6 (lane 1), 1D8, (lane 2), 4A7 (lane 3), 5A2 (lane 4), 5D8 (lane 5). The sizes of molecular weight standards are shown at the right (lane 6).

To determine the nature of the antigens recognized by the mAbs, total bacterial lysates were electrophoresed in non-reducing SDS-PAGE gels and probed by Western blotting. The mAbs 5A2 and 5D8 recognized a single antigen of ∼25 kDa. mAb 1C6 reacted with a major antigen of ∼16.5 kD and a minor immunoreactive antigen of ∼20 kD. mAb 4A7 recognizes a major antigen of ∼24 kD and a minor immunoreactive antigen of ∼28 kD. mAb 1D8 reacted with 3 antigens of different molecular weights (Fig. 2). Therefore, it suggested that hybridoma lines 5A2 (5D8), 1C6, 4A7, 1D8 produced antibodies against different localized antigens.

### Detection of E. coli O157:H7 from Food Samples by DIGFA

**Figure 3:**
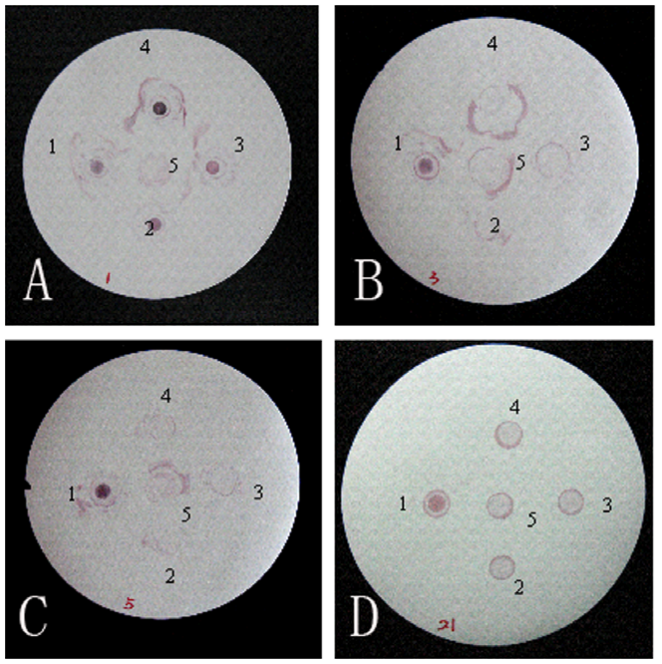
Detection of *E. coli* O157:H7 by the dot-immunogold filtration assay. Immune-colloidal gold probes coated with mAb 5A2 were applied to special single out *E. coli* O157:H7 contaminated minced beef from others which included food samples contaminated by ATCC 43895 (A1,B1,C1,D1), IHEM 1.3001 (A2), IHEM 1.3002 (A3), IHEM 1.3003 (A4), CMCC 50303 (B2), CMCC 50115 (B3), CMCC 50309 (B4), CMCC 51135 (C2), CMCC 51081 (C3), CMCC 51066 (C4), CMCC 44102 (D2), CMCC 44109 (D3), CMCC 44156 (D4). Negative control A5, B5, C5, D5) refers to food samples which have no bacterial contamination.

To further demonstrate the specificity of mAbs, 85 copies of minced beef contaminated by single different bacteria listed in the Table 1 were prepared and mAb 5A2 was used to detect *E. coli* O157:H7 by DIGFA method. Immune-colloidal gold probe coated with mAb 5A2 showed very good specificity and it singled out food samples contaminated by *E. coli* O157:H7 rapidly (within 3 minutes) and accurately (Fig. 3). mAb 5A2 distinguished *E. coli* O157:H7 specifically from 80 strains of *Salmonella sp.*, *Shigella sp*., *Proteus spp.*, *Yersinia enterocolitica*, *Staphylococcus aureus*, *Klebsiella pneumoniae*, *Citrobacter freundii* and other non-*E. coli* O157:H7 enteric bacteria.

To validate the sensitivity of this method, different concentrations of PBS-diluted *E. coli* O157:H7 which ranged from 10^5^ to 10^9^ CFU/mL was tested by DIGFA. This showed that its detection limit reached 5.5×10^6^ cfu/mL.

## Discussion

To detect food *E. coli* O157:H7 contamination rapidly and accurately, Cy-mediated S.I. strategy has been employed in the present study to obtain high specific mAbs 1D8, 4A7, 5A2.

The S.I. method has achieved the desired results in the production of mAbs of different cells and proteins in the past decade [6], [7], [8], [9], [19], [20], [21], [22], [23]. First, S.I. procedure provides a great new approach for immunization workers to prepare mAbs with high specificity. For example, Sakakibara *et al*. [7] found that the mAb 5H7-G1 generated by S.I. procedure could recognize egg antigens in the animal cortex of fertilized, but not unfertilized, *Xenopus* eggs. A S.I. strategy was also performed in rats to generate high specific mAbs that preferentially reacted with antigens on ureteric bud, but not adult inner medullary collecting duct cells [20]. Second, S.I. procedure demonstrated subtractive immunization may work as a strategy to uncover cellular events that operate during different cellular conditions of interest. Rasmussen and Ditzel [9] described a strategy producing a large panel of mAbs that recognized cell surface markers preferentially or exclusively expressed on metastatic vs. nonmetastatic cancer cells. Their study demonstrated the advantage of using the exquisitely discriminating recognition system of the immune system itself to scan the cell surface proteome for differentially expressed proteins. Third, S.I. procedure increased the percentage of specific mAbs evidently. It was reported by Ou *et al*. that the percentage of specific mAbs increased by 7.5 times with the Cy-induced immune tolerance, compared with the normal control group [24].

In general, the S.I. method was classified into three types according to the tolerance approaches for the host animals including high-zone tolerance, neonatal tolerance and drug tolerance [25]. Not only will different tolerance procedures produce various effects on the immune system of the host, but its subsequent antibody production mechanism was also different. Many studies have successfully taken the drug approach, especially Cy-mediated tolerance with exclusive reactivity to a desired antigen, over the past few years for its following advantages: (1) a mixture of tissue extracts can be used directly to immunize mice which avoids the cumbersome process of antigen purification and the loss of antigen activity; (2) mAbs can be prepared to distinguish between two structurally similar proteins or tissues; (3) it reduces the amount of tedious positive clone screening procedure; (4) Cy-mediated tolerization is a immune response that is easy to experimentally manipulate [26], [27].

So far, there is no report about S.I. application in mAbs production of bacteria. Although traditional hybridoma technology has been widely used to generate mAbs for bacteria, there still are some limitations in isolating specific antibodies for bacteria *e.g*. *E. coli* O157:H7 whose specific antigen is present in a complex mixture of proteins. Very few of the highly specific mAbs of *E. coli* O157:H7 were reported by the hybridoma strategy. On the other hand, the demand for highly specific mAbs of *E. coli* O157:H7 is increasing due to minute changes between species and its frequent outbreak in the past a couple of decades.

In this study, the method of S.I. by Cy-mediated tolerization was adopted to generate antibodies against *E. coli* O157:H7 for the first time. In general, donor mice are immunized first with the control antigens (*E. coli* O157:H19), immediately followed by treatment with the immunosuppressant drug, Cy. After these treatments, the mice would induce no immune response to the first antigens that act as tolerogens. Then, the same mice are immunized with the second antigens (*E. coli* O157:H7) that have very similar but distinct antigenic properties compared with the first antigens. According to this scheme, highly specific mAbs that preferentially reacted with antigens on *E. coli* O157:H7, but not *E. coli* O157:H19 are generated. This not only ovecomes the problem of the lack of specific antigen with high purity, but also increases the likelihood of producing mAbs against rare or weakly immunogenic epitopes, avoiding problems of poor immunogenic small-molecule protein and antibody instability. Furthermore, this procedure eliminates the production of undesired mAbs and helps generate some unique mAbs which have no cross-reaction with similar intestinal bacteria strains. In this study, the mAbs of 1D8, 4A7, 5A2 prepared by this strategy demonstrated no cross-reactivity with *Citrobacter freundii* and *Salmonella sp..* Furthermore, preparing specific mAbs by the S.I. procedure is cost-efficient and has high-throughput. In general, the traditional hybridoma technology was laborious and lacked for preparing mAbs of bacterium. Luciani *et al.* [28] prepared 101 mAbs of *E. coli* O157:H7 using standard immunization, but there were only 7 specific antibodies which reacted with *E. coli* O157:H7 only and not with the the 28 other bacterial antigens tested. In contrast, according to the methods of subtractive immunization, we screened 19 strains and found 3 mAbs of *E. coli* O157:H7 having no cross-reactivity with 80 non-*E. coli* O157:H7 bacteria. Its success ratio was 3/19, which was 3.8 times that of Lucinani's study [28].

In conclusion, highly specific mAbs against *E. coli* O157:H7 can be produced by the subtractive immunization technique in mice simply, rapidly and efficiently. Immune-colloidal gold probe coated with mAb 5A2 detects *E. coli* O157:H7 contamination from food samples rapidly and accurately by the DIGFA. Subtractive immunization may play a vital role in the generation of novel diagnostics mAbs in the future.

## References

[1] Watanabe Y, Ozasa K, Mermin J, Griffin P, Masuda K (1999). Factory outbreak of Escherichia coli O157: H7 infection in Japan.. Emerging Infectious Diseases.

[2] Navarro A, Eslava C, Garcia de la Torre G, Leon LA, Licona D (2007). Common epitopes in LPS of different Enterobacteriaceae are associated with an immune response against Escherichia coli O157 in bovine serum samples.. J Med Microbiol.

[3] Park CH, Martin EA, White EL (1998). Isolation of a nonpathogenic strain of Citrobacter sedlakii which expresses Escherichia coli O157 antigen.. J Clin Microbiol.

[4] Sowers EG, Wells JG, Strockbine NA (1996). Evaluation of commercial latex reagents for identification of O157 and H7 antigens of Escherichia coli.. J Clin Microbiol.

[5] Sleister HM, Rao AG (2002). Subtractive immunization: a tool for the generation of discriminatory antibodies to proteins of similar sequence.. J Immunol Methods.

[6] Villavedra M, Lemke S, To J, Broady K, Wallach M (2007). Carbohydrate epitopes are immunodominant at the surface of infectious Neoparamoeba spp.. J Fish Dis.

[7] Sakakibara K, Sato K, Iwasaki T, Kitamura K, Fukami Y (2005). Generation of an antibody specific to Xenopus fertilized eggs by subtractive immunization.. Genes Cells.

[8] Krueger P, Nitz C, Moore J, Foster R, Gelber O (2001). Monoclonal antibody identifies a distinctive epitope expressed by human multiple myeloma cells.. Journal of Immunotherapy.

[9] Rasmussen N, Ditzel HJ (2009). Scanning the cell surface proteome of cancer cells and identification of metastasis-associated proteins using a subtractive immunization strategy.. J Proteome Res.

[10] Westerman RB, He Y, Keen JE, Littledike ET, Kwang J (1997). Production and characterization of monoclonal antibodies specific for the lipopolysaccharide of Escherichia coli O157.. J Clin Microbiol.

[11] Lane R, Crissman R, Ginn S (1986). High efficiency fusion procedure for producing monoclonal antibodies against weak immunogens.. Methods in enzymology.

[12] McKinney MM, Parkinson A (1987). A simple, non-chromatographic procedure to purify immunoglobulins from serum and ascites fluid.. J Immunol Methods.

[13] Xin Z, Liu C, Dong B, Gao Y, Shao N (2004). A subtractive fluorescence-activated cell-sorting strategy to identify mimotopes of HBV-preS protein from bacterially displayed peptide library.. Journal of immunological methods.

[14] Loomans EE, Roelen AJ, Van Damme HS, Bloemers HP, Gribnau TC (1995). Assessment of the functional affinity constant of monoclonal antibodies using an improved enzyme-linked immunosorbent assay.. J Immunol Methods.

[15] Beatty JD, Beatty BG, Vlahos WG (1987). Measurement of monoclonal antibody affinity by non-competitive enzyme immunoassay.. J Immunol Methods.

[16] Wang X, Zhan W, Xing J (2006). Development of dot-immunogold filtration assay to detect white spot syndrome virus of shrimp.. J Virol Methods.

[17] Fukushima H, Katsube K, Hata Y, Kishi R, Fujiwara S (2007). Rapid separation and concentration of food-borne pathogens in food samples prior to quantification by viable-cell counting and real-time PCR.. Appl Environ Microbiol.

[18] Han FC, Hou Y, Yan XJ, Xiao LY, Guo YH (2000). Dot immunogold filtration assay for rapid detection of anti-HAV IgM in Chinese.. World J Gastroenterol.

[19] Joshi SA, Shaikh S, Ranpura S, Khole VV (2003). Postnatal development and testosterone dependence of a rat epididymal protein identified by neonatal tolerization.. Reproduction.

[20] Mernaugh RL, Yan H, Chen D, Edl J, Hanley G (2005). Production and characterization of mouse ureteric bud cell-specific rat hybridoma antibodies utilizing subtractive immunization and high-throughput screening.. J Immunol Methods.

[21] Lefebvre DJ, Costers S, Van Doorsselaere J, Misinzo G, Delputte PL (2008). Antigenic differences among porcine circovirus type 2 strains, as demonstrated by the use of monoclonal antibodies.. J Gen Virol.

[22] Ning Y, Wang Y, Li Y, Hong Y, Peng D (2006). An alternative strategy for high throughput generation and characterization of monoclonal antibodies against human plasma proteins using fractionated native proteins as immunogens.. Proteomics.

[23] Hooper JD, Zijlstra A, Aimes RT, Liang H, Claassen GF (2003). Subtractive immunization using highly metastatic human tumor cells identifies SIMA135/CDCP1, a 135 kDa cell surface phosphorylated glycoprotein antigen.. Oncogene.

[24] Ou SK, McDonald C, Patterson PH (1991). Comparison of two techniques for targeting the production of monoclonal antibodies against particular antigens.. J Immunol Methods.

[25] Holbrook F, Nicholson I, Zola H (2002). Tolerization as a tool for generating novel monoclonal antibodies.. Immunology and cell biology.

[26] Sleister H, Rao A (2001). Strategies to generate antibodies capable of distinguishing between proteins with> 90% amino acid identity.. Journal of immunological methods.

[27] Yang L, Wang W (2002). Preparation of monoclonal antibody against apoptosis-associated antigens of hepatoma cells by subtractive immunization.. WORLD JOURNAL OF GASTROENTEROLOGY.

[28] Luciani M, Armillotta G, Magliulo M, Portanti O, Di Febo T (2006). Production and characterisation of monoclonal antibodies specific for Escherichia coli O157:H7.. Vet Ital.

